# Failure of Ventricular Fibrillation Sensing During Subcutaneous Implantable Defibrillator Testing: A Twitchy Situation

**DOI:** 10.19102/icrm.2024.15112

**Published:** 2024-11-15

**Authors:** Mussa Saad, Deepti Ranganathan, Sheldon M. Singh

**Affiliations:** 1 Sunnybrook Health Sciences Centre, Toronto, Ontario, Canada; 2 Department of Medicine, Temerty Faculty of Medicine, University of Toronto, Toronto, Ontario, Canada

**Keywords:** Defibrillation threshold testing, diaphragmatic tetany, subcutaneous implantable cardioverter-defibrillator, undersensing

## Abstract

Oversensing of extra-cardiac noise may inhibit delivery of subcutaneous cardiac implantable defibrillator (S-ICD) therapy. We report a case of diaphragmatic tetany resulting in the inhibition of S-ICD therapy at the time of defibrillator testing without the use of muscle relaxants. Clinicians should be aware of this phenomenon.

## Case presentation

A 66-year-old man was admitted to our center following a syncopal event during which he sustained a C5 spinal cord injury. During his admission, he was referred for a subcutaneous cardiac implantable defibrillator (S-ICD) generator replacement. As such, we elected for conscious sedation during the S-ICD replacement procedure to avoid endotracheal intubation in the setting of the patient’s unstable cervical spine fracture.

**Figure 1:**
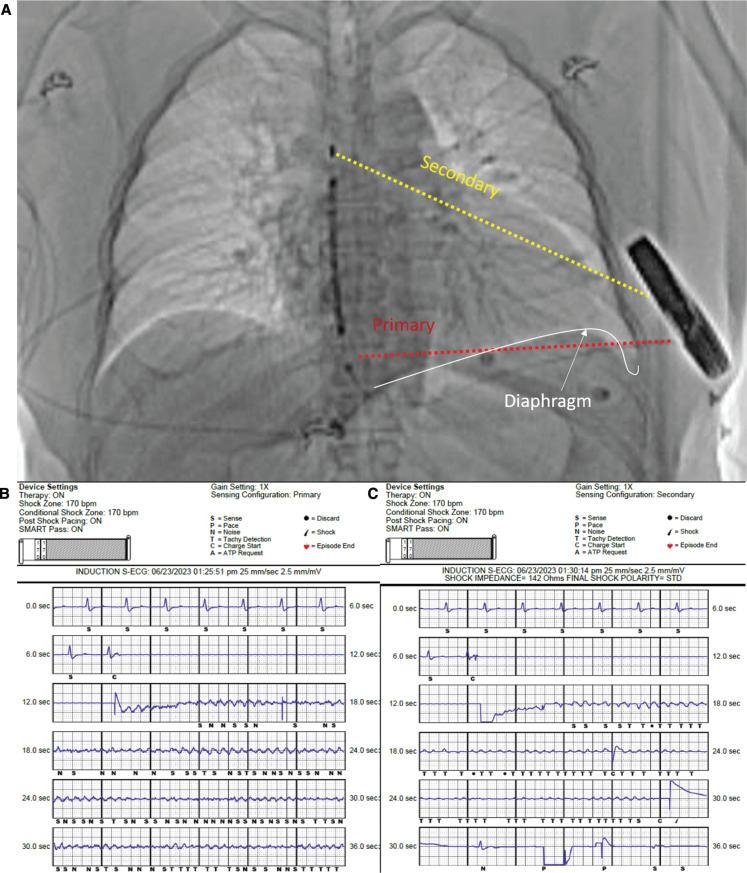
**A:** Location of the subcutaneous cardiac implantable defibrillator coil and can. The primary (red) and secondary (yellow) sensing vectors are shown. The left diaphragm is indicated in white and in the region of the primary sensing vector. **B:** Ventricular fibrillation (VF) induction in the primary sensing mode. Noise related to muscle tetany noted with an N preventing appropriate sensing indicated with a T. Criteria for the detection of VF not achieved at 36 s. **C:** VF induction in the secondary sensing mode. Appropriate T sensing with a charge committed at 8 s.

As per guideline recommendations, defibrillation threshold testing was performed.^1^ Prior to ventricular fibrillation (VF) induction, we flushed with saline to expel air from the incision sites, closed both incisions, and ensured that we tested for noise with massaging of the lead/generator. Using the Vector Select Algorithm (Boston Scientific, Marlborough, MA, USA), the device was programmed with a primary sensing vector **(Figure 1A)**. VF induction was performed with a 200-mA burst at 50 Hz for 5 s. VF was induced but not sensed appropriately by the device **(Figure 1B)**. After 30 s without delivery of therapy, an external shock was delivered. A review of the associated electrogram suggested inhibition of sensing due to the presence of noise. At this point, the sensing vector was manually programmed to the secondary vector, and VF induction was repeated. With this sensing configuration, noise was not detected **(Figure 1C)**. Sensing of VF was appropriate, and the therapy was committed 8 s into the episode and delivered 8 s later.

## Discussion

This case highlights the phenomenon of diaphragmatic tetany during VF induction with an S-ICD system. In the absence of the use of muscle relaxants, VF induction with a 50-Hz burst can stimulate adjacent extra-cardiac muscles—in this case, the diaphragm—resulting in muscular tetany. If the selected S-ICD sensing vector encompasses this muscle group, then the muscle activity can be sensed and interpreted as noise, thereby resulting in the therapy being withheld. Non-cardiac muscular tetany is unlikely to occur when muscle relaxants are used or when ventricular arrhythmia is induced with programmed electrical stimulation using a transvenous electrophysiology catheter.^2^ Furthermore, this phenomenon would not be expected to occur in the setting of spontaneous ventricular arrhythmia.

With the increasing use of S-ICD and the move away from the use of general anesthesia for S-ICD implantation, clinicians need to be aware of this phenomenon to avoid unnecessary repetitive VF induction or lead revision.
